# Predicting allosteric pockets in protein biological assemblages

**DOI:** 10.1093/bioinformatics/btad275

**Published:** 2023-04-28

**Authors:** Ambuj Kumar, Burak T Kaynak, Karin S Dorman, Pemra Doruker, Robert L Jernigan

**Affiliations:** Bioinformatics and Computational Biology Program, Iowa State University, Ames, IA 50011, United States; Roy J. Carver Department of Biochemistry, Biophysics and Molecular Biology, Iowa State University, Ames, IA 50011, United States; Computational Neurobiology Laboratory, Salk Institute for Biological Studies, La Jolla, CA 92037, United States; Department of Computational and Systems Biology, School of Medicine, University of Pittsburgh, Pittsburgh, PA 15232, United States; Bioinformatics and Computational Biology Program, Iowa State University, Ames, IA 50011, United States; Department of Statistics, Iowa State University, Ames, IA 50011, United States; Department of Computational and Systems Biology, School of Medicine, University of Pittsburgh, Pittsburgh, PA 15232, United States; Bioinformatics and Computational Biology Program, Iowa State University, Ames, IA 50011, United States; Roy J. Carver Department of Biochemistry, Biophysics and Molecular Biology, Iowa State University, Ames, IA 50011, United States

## Abstract

**Motivation:**

Allostery enables changes to the dynamic behavior of a protein at distant positions induced by binding. Here, we present APOP, a new allosteric pocket prediction method, which perturbs the pockets formed in the structure by stiffening pairwise interactions in the elastic network across the pocket, to emulate ligand binding. Ranking the pockets based on the shifts in the global mode frequencies, as well as their mean local hydrophobicities, leads to high prediction success when tested on a dataset of allosteric proteins, composed of both monomers and multimeric assemblages.

**Results:**

Out of the 104 test cases, APOP predicts known allosteric pockets for 92 within the top 3 rank out of multiple pockets available in the protein. In addition, we demonstrate that APOP can also find new alternative allosteric pockets in proteins. Particularly interesting findings are the discovery of previously overlooked large pockets located in the centers of many protein biological assemblages; binding of ligands at these sites would likely be particularly effective in changing the protein’s global dynamics.

**Availability and implementation:**

APOP is freely available as an open-source code (https://github.com/Ambuj-UF/APOP) and as a web server at https://apop.bb.iastate.edu/.

## 1 Introduction

Allosteric regulation of function is commonly observed in proteins, ranging from the small G proteins (Mott and Owen 2018) to larger assemblies such as GroEL during its chaperoning of folding (Lin and Rye 2006) and even to microtubules during transport (Amos and Löwe 2014). Conformational transitions, such as those between open/closed and on/off states of proteins, routinely occur during allosteric events of regulation, i.e. activation, inhibition, or more subtle control of function. Binding of ligands (small or large) at an allosteric site alter protein conformations and/or dynamics, can thereby control binding events at distant, functional site(s) (Zhang et al. 2020). The current understanding of allostery is based on conformational dynamics and selection mechanisms. In this perspective, allosteric regulation can take place even in the absence of significant changes to the protein conformation (Popovych et al. 2006; Daily and Gray 2007; Tsai et al. 2008), in which case the change in protein dynamics becomes the main mechanism for regulation. In particular, the so-called global or collective motions that are essential for protein function have been shown to be significantly altered during ligand binding and allosteric events (Kaynak and Doruker 2019; Kaynak et al. 2020).

The large-scale transitions observed over long times (Leioatts et al. 2012; Gur et al. 2013) are not so readily accessible by molecular dynamics simulations, and this means there is a need for computationally efficient approaches facilitated by the use of coarse-grained models. From this perspective, the elastic network models (ENM) (Bahar et al. 1997; Atilgan et al. 2001) are appropriately used to efficiently represent the functional protein dynamics (Yang et al. 2007; Katebi and Jernigan 2014; Zimmermann et al. 2016; Mishra et al. 2017; Mishra and Jernigan 2018; Kumar and Jernigan 2021), especially for large assemblages. Specifically, the low-frequency or global modes obtained from the ENM vibrational spectra are known to guide the large-scale allosteric transitions (Tama and Sanejouand 2001; Yang et al. 2007; Katebi and Jernigan 2014; Zimmermann et al. 2016; Mishra et al. 2017). ENMs applied to a large dataset of small ligand-protein complexes have shown that ligand binding introduces new constraints on the global modes (Kaynak and Doruker 2019). Based on this insight an ENM-based methodology, named Essential Site Scanning Analysis (ESSA), was recently introduced for identifying the so-called essential sites that can significantly modify global modes, at allosteric ligand-binding sites and hinges (Kaynak et al. 2020). In addition, Kaynak et al. (2020) and Song et al. (2017) showed that the allosteric pockets within a protein tend to have a higher hydrophobicity compared to other pockets in the same protein. The hydrophobic nature of allosteric pockets plays a role in the binding of small, hydrophobic molecules, which can modulate the protein's activity. This characteristic helps to create a specific and favorable environment for the binding of allosteric ligands, leading to changes in the protein's conformation and function. Therefore, it allows for the identification of highly probable allosteric pockets with greater hydrophobicity, which is also crucial for drug targeting. Other diverse computational approaches have been utilized for allosteric site prediction and signal transfer, ranging from molecular dynamics simulations (Hacisuleyman and Erman 2017; Singh and Bowman 2017), to normal mode analyses (Zheng et al. 2007; Balabin et al. 2009; Mitternacht and Berezovsky 2011; Panjkovich and Daura 2012; Rodgers et al. 2013) and graph theory (Amor et al. 2016), as well as machine learning approaches (Greener and Sternberg 2015; Song et al. 2017; Mishra et al. 2019; Ferraro et al. 2021; Marchetti et al. 2021).

In this study, we simulated the impact of ligand binding on the global mode of proteins by considering pockets identified by the Fpocket algorithm (Le Guilloux et al. 2009). While binding of substrate to active sites can also lead to large change in the global modes of proteins, these pockets generally tend to have relatively more polar residues. Previous studies showed that allosteric pockets are relatively more hydrophobic than other pockets in a protein. This distinction helps to differentiate allosteric pockets from all other pockets, including active site pockets. However, not all hydrophobic pockets have the ability to alter global motions. With this in mind, we developed a new Allosteric Pocket Prediction method (APOP) that considers a combination of frequency shifts in the global modes obtained from perturbations applied to Gaussian network models (GNM) (Bahar et al. 1997; Haliloglu et al. 1997) for protein dynamics, and the local hydrophobic density obtained from Fpocket to accurately identify allosteric pockets in proteins. APOP is a pocket-based algorithm, which utilizes Fpocket (Le Guilloux et al. 2009) for locating existing pockets in a protein structure. Results of applying APOP to a diverse set of 104 proteins, both monomeric and multimeric, indicates a high success rate, where the known allosteric pockets are predicted within the top 3 in the rank of the 92 proteins. APOP outperforms machine learning methods Allopred (Greener and Sternberg 2015) and Passer (Tian et al. 2021), which have been shown to predict known allosteric pockets with relatively high accuracy (see Section 3).

Larger numbers of important allosteric pockets are found. In several cases, we show that APOP can predict alternative allosteric pockets, as well as successfully utilize alternative conformers, such as apo or holo structures. Other important allosteric pockets discovered are the large pockets commonly found at the centers of multimeric assemblies.

## 2 Materials and methods

### 2.1 Dataset

The allosteric proteins used in this study are taken from the test set of Allopred (Greener and Sternberg 2015), together with the apo/holo structures used in ESSA (Kaynak et al. 2020), as well as some additional cases from a recent literature search (see Supplementary Tables S1–S5). Multi-chain protein assemblages are generated with PyMol (Schrödinger) according to the information provided in the Protein Databank (PDB) (Berman et al. 2000). In total, we have a set of 61 monomers and 43 multimeric structures (see Supplementary Tables S1–S5). The number of pockets in this set ranges from 10 to 242 (Supplementary Table S1). We consider ranks 1, 2, and 3 as successes in predicting known allosteric pockets across the large range of known pockets in proteins (see Supplementary Tables S1–S5).

### 2.2 APOP

Our new allosteric pocket prediction algorithm APOP is comprised of three steps: (i) Pocket hunting. Pockets in the input protein structure are identified by Fpocket protein cavity detections (Le Guilloux et al. 2009), which uses Voronoi tessellation and alpha shapes to identify each pocket. This step is carried out using the default parameters of Fpocket. (ii) Perturbation of pockets. Each pocket in the protein elastic network (GNM) is perturbed by inserting stiffer springs between the residues lining the pocket under consideration. (iii) Scoring. The pockets are scored and ranked according to the computed eigenvalue shifts in the global GNM modes together with their local hydrophobic densities (a feature from Fpocket). More details about the 2nd and 3rd steps are provided next.

#### 2.2.1 Perturbation of pockets

GNM is the underlying dynamics model used here to obtain a measure of the impact of perturbations at a given pocket on the protein’s characteristic motions, i.e. the global modes (Bahar et al. 1997). GNM has recently been shown to yield the essential residues that affect the global modes and was previously shown to be adept at predicting the allosteric pockets (Kaynak et al. 2020) for monomeric proteins. GNM uses a coarse-grained approach, where the alpha carbon ($C^{\alpha })$ atoms in a structure are chosen as the nodes in the elastic network. Here, the total potential energy is the sum over the harmonic potentials connecting residue pairs i and j, with any displacements from the original structure considered to be higher in energ*y* where *N* is the total number of residues/nodes in the protein. $\Delta R_{i}$ and $\Delta R_{j}$ represent the corresponding displacements of residues i and j from their equilibrium positions. There is a uniform spring constant, $\gamma =1.0\;\mathrm{kcal}\;{\mathrm{mol}}^{-1}\;{Å}^{-2},$ between all pairs of i and j nodes.


V=12γ∑i, jNΓijΔRi-ΔRj2 (1)

The connectivity matrix or contact map, Γ, determines the placement of springs between neighboring residue pairs if they lie within a cutoff distance of 10 Å.


$$\begin{array}{l} \Gamma_{ij}=\left\{\begin{array}{l} -1\;\;\;\;\;\;\;\;\;\;\;\;\;\;\mathrm{if}\;i\neq j\;\mathrm{and}\;R_{ij}\leq 10\;Å\\ \;0\;\;\;\;\;\;\;\;\;\;\;\;\;\;\;\mathrm{if}\;i\neq j\;\mathrm{and}\;R_{ij}>10\;Å\\ -\sum_{i,\;i\;\neq j}\Gamma_{ij}\;\;\mathrm{if}\;i=j\; \end{array}\right.\; \end{array}\left(2\right)$$

Here, $R_{ij}$ is the distance between residues i and j at their equilibrium positions in the reference structure. The eigenvectors (u**)** and eigenvalues (λ) of the contact map of this reference structure are obtained by singular value decomposition of Γ following removal of the rigid body mode of motion.

To mimic the effect of ligand binding to a specific pocket, we increase the spring constant between all residues participating in that pocket, where γ is set to 10.0 kcal ${\text{mol}}_{\text{−}}^{1}$ Å−^2^ regardless of the distances between those pairs of residues. For each perturbed pocket, the eigenvalues, and eigenvectors are calculated for the whole new structure network.

#### 2.2.2 Scoring

After perturbing each pocket one-by-one, we score the constraining effect thereof on protein global motions by adopting a similar measure that was developed for ESSA (Kaynak et al. 2020). This measure is based on a comparison between the eigenvalues of the perturbed and unperturbed structures. This comparison requires a prior matching between the global modes as possible shifts in the mode indices may occur due to perturbations. For this purpose, we first calculate the overlap matrix between the slowest 5 eigenvectors of the unperturbed structure ($v_{m}$**)** and the first 15 eigenvectors of the perturbed structure ($v_{n}^{p}$) a*s* where *m* and *n* refer to the indices of the modes. The paired indices of the perturbed modes are reassigned based on the best overlaps. This gives a set of reordered first 5 most important modes for the perturbed structure.


overlapmn=vm.vnpvmvnp 3

Once we have the matching modes, we can evaluate the percentage shift in λ for these matched modes in response to the perturbation of a specific pocket p


Δλkp=λkp-λkλk×100, (4)

Here $\lambda_{k}$ is the *k*th eigenvalue obtained for the unperturbed/reference structure and $\lambda_{k}^{p}$ is the corresponding eigenvalue obtained for the perturbed structure. Changes in global modes are determined by the mean percentage eigenvalue shifts over the first five modes (1 ≤ *k* ≤ 5), $〈{\Delta \lambda }^{p}〉.$ This part of the scoring was originally developed in our ESSA method (Kaynak et al. 2020). Afterwards, a *z*-score $z_{p}$ is assigned to each pocket to assess the effect of ligand binding at that specific pocket *p* on the global dynamics b*y*


zp=Δλp-μσ (5)

Here, μ and σ denote the respective mean and the standard deviation of $〈{\Delta \lambda }^{p}〉$ over all pockets.

Allosteric pockets were previously shown to have higher local hydrophobic density (Song et al. 2017), $H_{p}$, which is also a feature calculated by Fpocket. Use of $H_{p}$ was shown to improve allosteric pocket prediction in ESSA (Kaynak et al. 2020). The *z*-scores of the pocket local hydrophobic densities ($z_{hp}$**)**, are calculated usin*g*


zhp=Hp-μhσh (6)

Here, $\mu_{h}$ and $\sigma_{h}$ are, respectively, the mean and standard deviation of $H_{p}$ over all pockets in the protein. These *z*-scores are then combined to define a pocket allostery score, giving equal weight to each of the two considerations as,


sp=zp+zhp2 (7)

Then, the pockets are ranked based on this combined score $s_{p}$, which characterizes the relative allosteric propensity of each pocket.

### 2.3 Allopred and passer

For benchmarking, we use two machine learning-based methods, Allopred (Greener and Sternberg 2015), and Passer (Tian et al. 2021), which had significant successes in allosteric pocket predictions. Both Allopred and Passer pocket features are obtained from Fpocket. In addition, Allopred combines dynamics information from ENM (changes in the deformation of active site residues resulting from the perturbation in the pockets).

### 2.4 Success criteria

For each structure, we report the highest-ranked pocket that is known to have bound allosteric ligand(s). In the multimeric assemblages, the same allosteric ligand(s) can be bound to each subunit. Among those occurrences, we report the highest-ranking pocket observed in the multimer. If this pocket is among the top-ranked three predicted pockets, we count it as a success. To cross-validate our results for APOP, Allopred, and Passer performance, we visually check whether the known allosteric ligand resides within the three top-ranked pockets reported by each method.

### 2.5 Statistical analysis

A one-sided Wilcoxon signed-rank test (Obremski and Conover 1981) was applied to test if there is a significant difference between known allosteric pocket ranking performance between APOP and Allopred. Here, the Null hypothesis H_0_ is: The median difference between rank of known allosteric pocket predicted using Allopred and APOP is zero. The contrasting Alternate hypothesis H_1_ is: The median difference between rank of known allosteric pocket predicted using Allopred and APOP is positive. A positive difference indicates that the predicted rank of known allosteric pocket with Allopred is greater than APOP. A thorough statistical comparison between APOP and Passer was not conducted since the Passer web server provides only the top three ranking pockets, which leads to many unranked pockets.

## 3 Results and discussion

### 3.1 APOP successfully predicts allosteric pockets in holo-structures

APOP’s performance in predicting allosteric pockets is first tested on a dataset of 50 protein structures with bound allosteric ligands (Supplementary Table S1). Our allosteric pocket predictions are based on holo-structures formed simply by removing any ligand(s). We apply both APOP and Allopred to each crystal structure and report the ranks in Supplementary Table S1. Allopred requires active site residue information for allosteric pocket prediction; thus, pockets located at the active site(s) are removed from the rankings to permit a more direct comparison between APOP and Allopred. Our results indicate that APOP outperforms Allopred and Passer in predicting the known allosteric pockets in the dataset. APOP is able to detect the allosteric pockets at first rank for 35 proteins in contrast to 19 proteins for Allopred and 29 for Passer (Supplementary Table S1). If we consider the top 3 ranked pockets, the overall success rate for APOP is 84% (42/50), surpassing that of Allopred at 68% (34/50) and Passer at 76% (38/50). Furthermore, the *P*-value of 0.00088 obtained from the one-sided Wilcoxon signed-rank (Obremski and Conover 1981) results indicate the ranking of known allosteric pocket obtained with APOP to be significantly better than with Allopred.

We emphasize that APOP does not require prior knowledge of active site residues for predictions, unlike Allopred. Specifically, Allopred could not be applied to the Acyl-coenzyme A thioesterase 11 protein crystal structure (PDB ID: 6VVQ) nor to Nuclease SbcCD subunit D (PDB ID: 6ASC), since the active site residues were not resolved for these crystal structures, because of missing residues. In contrast, APOP predicts the allosteric pockets at the number one place for both cases. Furthermore, APOP was only able to rank 32% (16/50) of the active site pockets as top ranked pockets (Supplemental Table S1), indicating that the APOP score-based pocket ranking does prioritize allosteric pockets over active site pockets.

### 3.2 Allosteric pockets can be predicted in different states including apo

We also assess the performance of APOP on *apo* structures, with the ligand-binding pockets either formed or stabilized by the ligand. For this purpose, we consider another dataset, of 15 proteins taken from Kaynak et al. (2020) and added structures from an additional literature search, where both *apo* and *holo* structures are available (Supplementary Table S2), so that we can detect the allosteric pockets in the *apo* structures. APOP successfully predicts allosteric pockets in all *holo*-structures (15/15) and 11 out of 15 pockets in *apo* structures in this set within the top 3 rank. Although conformational rearrangements seem to affect the success rate of our predictions for *apo* structures, we still achieve a satisfactory prediction rate of 86% (11/14), excluding one cryptic pocket (PDB ID: 1ZG4). For only the top-ranked pockets, APOP is successful in 12 *holo* and 8 *apo* structures.

**Figure 1.**
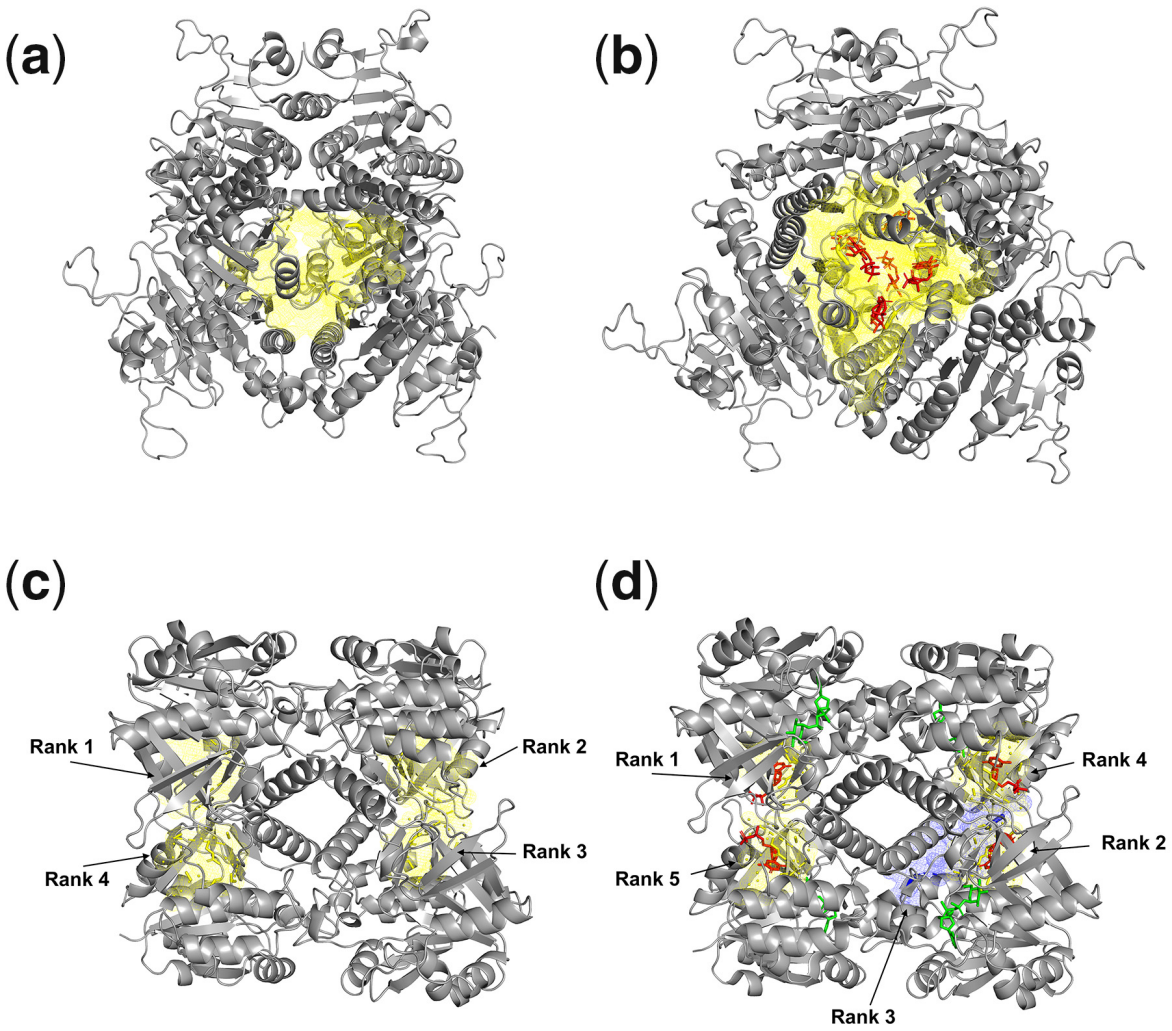
Validation by APOP for known allosteric pockets in uridylate kinase and glucose-1-phosphate thymidylyltransferase structures, for both apo and holo conformations. (a) apo form of uridylate kinase (PDB ID: 3EK6) where a known allosteric pocket is predicted as the rank 1 pocket by APOP from among a total of 88 pockets in the structure, (b) holo form of uridylate kinase (PDB ID: 3EK5) where the known allosteric pocket is predicted as rank 1 pocket by APOP from among the 84 pockets in the structure. (c) apo state of glucose-1-phosphate thymidylyltransferase (PDB ID: 1FZW) where the known allosteric pocket is predicted as the rank 1 pocket by APOP from among the total of 66 pockets in the structure, (d) holo state of glucose-1-phosphate thymidylyltransferase (PDB ID: 1H5T) where the known allosteric pocket is predicted as the rank 1 pocket by APOP from the total of 60 pockets in the structure. Allosteric ligands are shown in red, and the corresponding allosteric pockets predicted by APOP are shown in yellow. Substrates are colored green.

Figure 1 shows two results from the *apo*/*holo*-structure dataset. Uridylate kinase is an essential enzyme for pyrimidine biosynthesis, adding a phosphate to uridine monophosphate (UMP) to form uridine diphosphate (UDP) (Tu et al. 2009). It forms a homo-hexameric assembly, where six GTP molecules bind to its central cavity and act as allosteric effectors, causing a long-range allosteric response (Tu et al. 2009). APOP predicts the large central cavity as the top-ranked allosteric pocket in both the *apo* (PDB ID: 3EK6) and *holo* (PDB ID: 3EK5) structures (Fig. 1a and b). Moreover, APOP ranks all four allosteric ligand-binding pockets as top ranked pockets in both the *apo* and the *holo* conformational states for Glucose-1-phosphate thymidylyltransferase (Fig. 1c and d).

To further test APOP performance, a combination of native, mutant, open, and closed states, as well as *apo* and *holo* states of Tyrosine-protein phosphatase non-receptor type 1 protein are explored. Our results show that APOP is able to predict the known allosteric pocket as the top rank 1 pocket in 12, rank 2 in one case, and rank 5 in one structure from the set of 14 different conformational states (Supplementary Table S4).

### 3.3 Central cavities in protein assemblies often have a high proclivity to be allosteric

**Figure 2.**
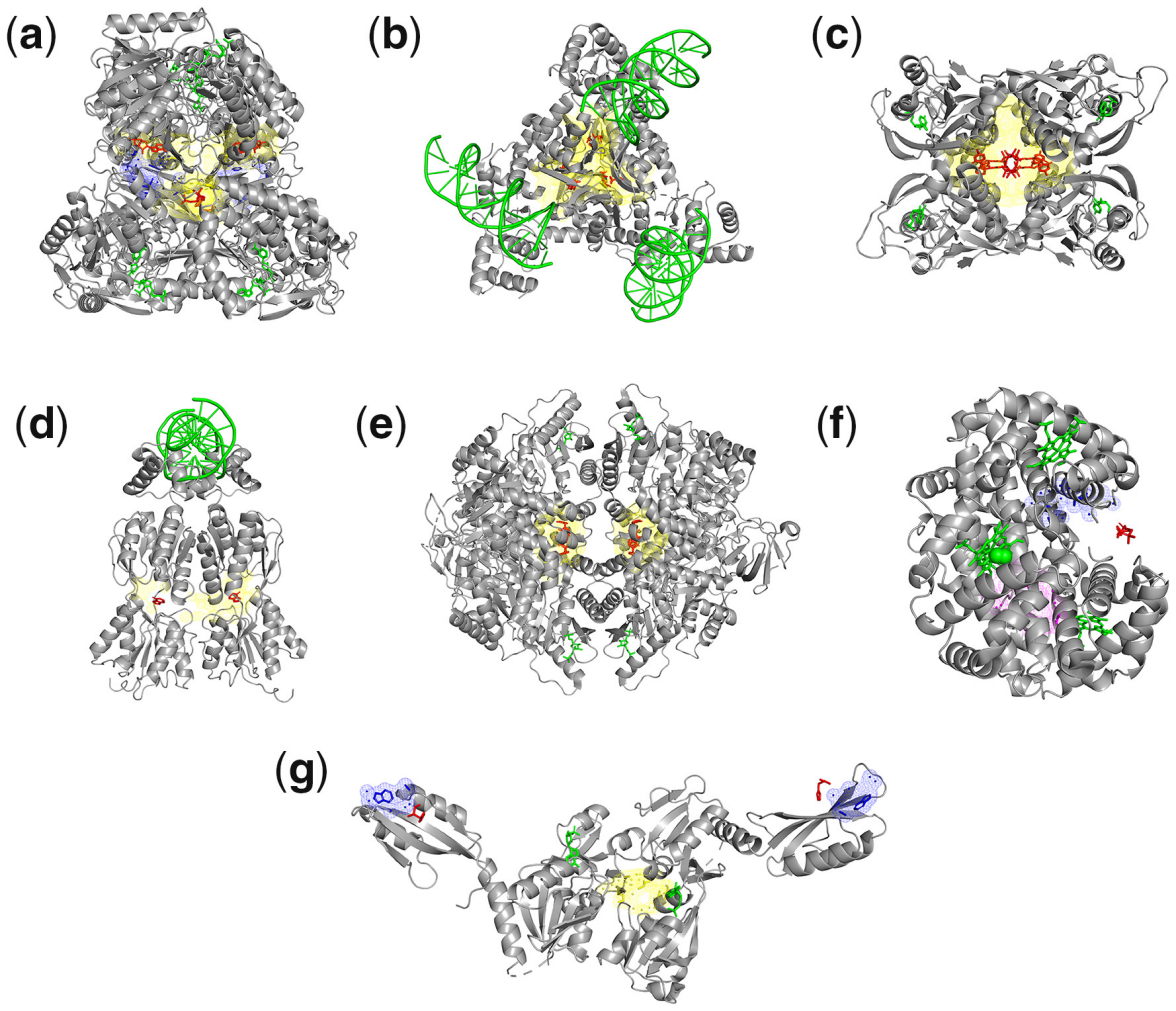
Allosteric pockets at the centers of molecular assemblages predicted by APOP. Resolved allosteric ligands are shown in red, and the corresponding allosteric pockets predicted by APOP are shown in yellow. Substrates are colored green. (a) In tetrameric glyceraldehyde-3-phosphate dehydrogenase (PDB ID: 1UXV), there are four identical allosteric ligands bound, among which the top-ranking allosteric pocket is ranked 7 among the total of 114 pockets in the tetrameric structure, (b) arginine repressor (PDB ID: 3LAJ) (allosteric pocket rank 1), (c) uracil phosphoribosyl transferase (PDB ID: 1XTU) (allosteric pocket rank 1), (d) *Escherichia coli* purine nucleotide synthesis repressor (PDB ID: 1QP0) (allosteric pocket rank 1), (e) pyruvate kinase M2 (PDB ID: 3H6O) (allosteric pocket rank 1), (f) hemoglobin (PDB ID: 1B86), where the known allosteric ligand-binding region was not predicted as a pocket by Fpocket, and therefore it was not ranked by APOP. Here, the central pocket (magenta mesh) has rank 1. The pocket, shown as blue mesh, is the closest to the allosteric ligand but does not include it. (g) ATP phosphoribosyl transferase (1NH8), where the Fpocket fails to detect a pocket where allosteric ligand binds. Here, Fpocket predicts a pocket (shown as blue mesh) near the ligand-binding region, but it does not enclose the ligand.

Large central cavities are observed to act as allosteric pockets in multimeric assemblages in our dataset, especially in homo-oligomeric cases, such as the top-ranked pocket just shown for uridylate kinase (Fig. 1a and b). Other top-ranked central pockets are illustrated in Fig. 2 for glyceraldehyde-3-phosphate dehydrogenase (ranked 7), arginine repressor (ranked 1), uracil phosphoribosyltransferase (ranked 1), and purine nucleotide synthesis repressor (ranked 1) (Supplementary Table S1). Perturbation of these central pockets often has a major effect on the global modes, limiting the large-scale inter-subunit motions and probably imposing particularly strong allosteric control. As central cavities are physically connected with many protein assembly subunits, they have a potential for being especially important effectors of the global motions of the assembly and would have significantly larger effects than other pockets. We should also note that considering these in druggability simulations could help to guide drug design and pharmacophore modeling studies.

We next focus on Pyruvate kinase M2, where binding of two identical activators to its central pocket were shown to aid in suppressing tumor growth (Anastasiou et al. 2012). APOP ranks the central allosteric activator binding pocket as the top pocket (Fig. 2e) in six human Pyruvate kinase M2 structures (Supplementary Table S3), as well as in two *Trypanosoma cruzi* Pyruvate kinase structures.

Hemoglobin (Fig. 2f) binds to the allosteric ligand 2,3-diphosphoglyceric acid in a region, which is not a central cavity as in the other cases presented here. In fact, the position of this ligand is stabilized by loops, which does not correspond to a cavity. Therefore, Fpocket fails to predict this as a pocket, and leads to failure by APOP and other methods (Supplementary Table S1) to predict it as an allosteric site. The closest pocket to the ligand is shown in blue mesh. However, there is another pocket located in the central cavity of hemoglobin tetramer (magenta mesh, shown from the side), which has the top rank excluding the top pockets that correspond to the heme ligand-binding regions in the structure. ATP phosphoribosyl transferase binds to histidine, which acts as an inhibitor, regulating histidine biosynthetic pathway through a feedback mechanism (Cho et al. 2003). Here, Fpocket fails to predict pocket in ATP phosphoribosyl transferase where histidine binds, and therefore APOP fails to rank the known histidine binding region as an allosteric pocket (Fig. 2g). Interestingly, similar to other assemblages shown in Fig. 2, APOP predicts a central pocket as rank 1 pocket (Fig. 2g, shown in yellow) in ATP phosphoribosyl transferase which can be an allosteric pocket.

### 3.4 APOP can predict alternative allosteric pockets

**Figure 3.**
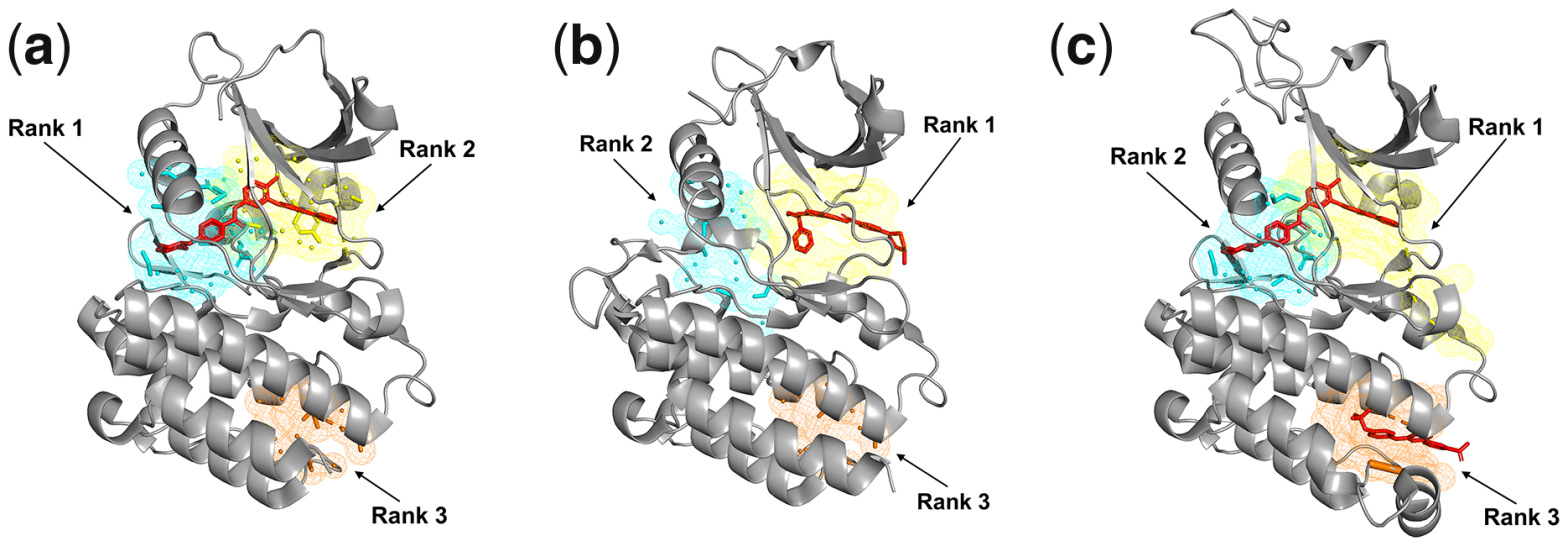
The three allosteric pockets are predicted as top-ranked pockets with APOP for ABL kinase (pockets shown in yellow). Three ABL kinase crystal structures are shown that are in complexes with: (a) imatinib (PDB ID: 2HYY) where the known allosteric ligand-binding pocket is predicted as rank 1 and rank 2 pocket by APOP among the total of 14 pockets in the structure, (b) the inhibitor PHA-739358 (PDB ID: 2V7A) where known allosteric ligand-binding pocket is predicted as rank 1 pocket by APOP from among the total of 15 pockets in the structure, (c) imatinib and GNF-2 (PDB ID: 3K5V) where the three known allosteric ligand-binding pockets are predicted as the ranked 1, 2, and 3 pockets by APOP among the total of 14 pockets in the structure.

Different allosteric pockets can be resolved in complexes of the same or homologous protein(s) bound with alternative allosteric ligands and APOP can predict such alternative pockets. The first example is ABL kinase, which plays an important role in cell growth and survival through a wide range of molecular functions such as cell motility, autophagy, apoptosis, remodeling of cytoskeleton, and receptor endocytosis (Umezawa and Kii 2021). Thus, ABL kinase has been widely studied for the design of selective allosteric inhibitors. Figure 3 shows three crystal structures in complex with different inhibitors (see details in Supplementary Table S5). In Fig. 3a, the first and second ranked pockets have bound imatinib, whereas a smaller inhibitor (PHA-739358) is bound to the top-ranking pocket in Fig. 3b. In another structure, imatinib again is bound to the same pockets (at first and second rank) and a second inhibitor (GNF-2) is shown in an alternative pocket (third rank). Thus, APOP can predict these alternative allosteric pockets in ABL kinase.

**Figure 4.**
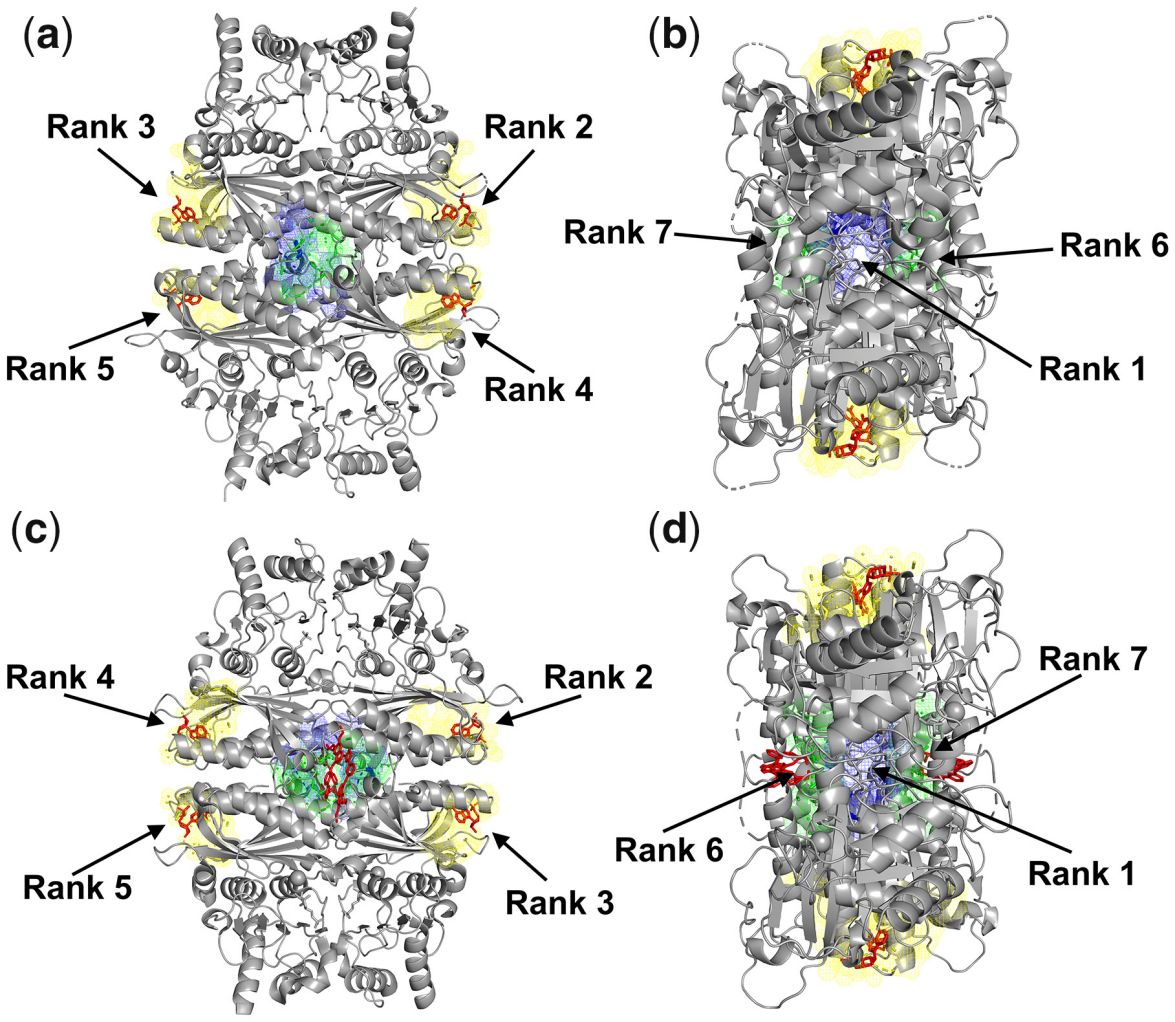
APOP finds allosteric pockets as top-ranked pockets in two homologous structures. (a and b) Human fructose-1,6-bisphosphatase structure (PDB ID: 3IFA) with a total 84 pockets in the assemblage. Here, APOP rank 2, 3, 4, and 5 pockets overlap with known allosteric pockets shown in yellow, rank 6 and 7 pockets are in green, and the rank 1 pocket is in blue. (c and d) *S.scrofa* fructose-1,6-bisphosphatase structure (PDB ID: 1KZ8) has a total of 69 pockets in its assemblage. APOP ranked 2, 3, 4, and 5 pockets overlap with known allosteric pockets reported in the structure and are shown in yellow, rank 6 and 7 pockets are shown in green, and rank 1 pocket in blue. The known allosteric ligands are in red.

The second example is fructose-1,6-bisphosphatase (F16Pase), which catalyzes the hydrolysis of fructose-1,6-bisphosphate (F16P) to fructose-6-phosphate (F6P). This homo-tetrameric enzyme plays an important role in regulating gluconeogenesis as a primary control point and helps maintain blood glucose levels. Figure 4 shows two homologous F16Pase structures bound to allosteric inhibitors. F16Pase is known to be regulated by adenosine monophosphate (AMP), which acts as an allosteric inhibitor (Zarzycki et al. 2011). In human F16Pase (Fig. 4a and b), APOP ranks the AMP-binding allosteric pockets at ranks 2, 3, 4, and 5 (shown in yellow mesh, with one pocket in each subunit). Next, we focus on a homologous F16Pase structure from *Sus scrofa* (PDB ID: 1KZ8) (Wright et al. 2002) (Fig. 4c and d), which is bound to AMP and another allosteric inhibitor (PFE, an anilinoquinazole). We observe a similar ranking of the central pocket (rank 1), followed by the AMP-binding pockets (ranks 2–5, green). Interestingly, the next two pockets (ranks 6 and 7) in both species correspond to the PFE- binding site observed in *S.scrofa*. Even though we concentrate on the top three-ranked pockets in our analysis, additional pockets with high rank may also have allosteric potential in multimeric structures that bind to multiple ligands. Notably, in some cases, there are more than 100 pockets detected by Fpocket in large assemblies. The total number of pockets found in F16Pase is 84 (PDB ID: 3IFA) and 69 (PDB ID: 1KZ8)—so that concentrating on the top 10% ranking pockets would include all of the allosteric pockets mentioned above. APOP also predicts the top-ranked pocket at the center of the assembly (blue mesh) in both species. In line with other cases discussed (see Fig. 2), this central pocket has the potential to serve as an allosteric pocket for drug design. Prediction of such novel pockets may help to design more effective allosteric ligands to regulate protein function in a species-specific way since these pockets might not have all details fully conserved across all species.

### 3.5 Implementation

APOP is available as an open-source Python package (https://github.com/Ambuj-UF/APOP), as well as on a user-friendly web interface (https://apop.bb.iastate.edu/). Here, the user can choose either to provide a PDB id or upload a protein structure and can add a specific chain id of interest, where APOP uses all chains present in the structure. APOP uses the default optimal GNM distance cutoff of 10.0 Å (see Section 3), but the web interface allows users to select the cutoff value.

## 4 Conclusion

The extent of dynamic changes to global modes upon perturbing the identified pockets, together with their local hydrophobicity scores, have demonstrated a high efficiency in predicting allosteric pockets across many proteins. Here, APOP is demonstrated to predict known allosteric pockets within the top ranked 3 pockets in a total 92 out of 104 (88.5%). APOP can predict allosteric pockets in both *apo* and *holo* structures, as well as in various mutant conformational states. We also show that APOP can accurately predict allosteric pockets in monomers as well as large macromolecular assemblages. Moreover, APOP can also detect alternative allosteric pockets as high-ranked pockets, indicating its potential utility for designing ways to alter protein activity by targeting newly identified ligand-binding pockets. Prediction of alternate allosteric pockets can facilitate the effective drug targeting of enzymes such as Phosphofructokinase, Glyceraldehyde-3 phosphate dehydrogenase, and Pyruvate kinase (Ayyildiz et al. 2020). APOP can also predict the known allosteric ligand-binding pockets as the top-ranked pockets in different protein conformational states. It is a useful tool to identify the most relevant allosteric pocket(s) for drug design and will reduce the time and investment required for drug design. One interesting result from this study is the finding that large central pockets are likely to be particularly effective allosteric binding sites since they are sites where ligand binding could have especially largest effects on protein dynamics. Also, the discovery of significant numbers of allosteric binding sites for many proteins reveals the complexity and the high potential for multiple levels of control, revealing possible details of multiple modes of control with the potential to regulate control in many different ways.

By comparing the APOP-predicted pockets to annotated pockets in the literature, we found that pockets that ranked high in our predictions were likely to be allosteric, even if they were not previously annotated as such. This is illustrated by the examples of fructose-1,6-bisphosphatase and pyruvate kinase (Fig. 4), where APOP was able to predict the central pocket as the top-ranked allosteric pocket, despite some of the crystal structures not having this pocket annotated as allosteric pocket in the corresponding literature. Furthermore, our analysis of false negatives showed that the known allosteric pockets that were not ranked among the top three were, but typically ranked within top 10 in large multimeric assemblages, are also known allosteric pockets, indicating that these pockets are also worth exploring. Our method's dependence on the Fpocket algorithm may be a limitation in some cases, such as in the ATP phosphoribosyltransferase (PDB ID: 1NH8) and Hemoglobin (PDB ID: 1B86) structures, where Fpocket failed to predict a pocket in the allosteric ligand-binding region. Nonetheless, our results suggest that APOP has the potential to be a valuable tool for future allosteric ligand discovery.

## Supplementary Material

btad275_Supplementary_Data
