# Supplementary material for: Predicting allosteric pockets in protein biological assemblages
Source: Bioinformatics. 2023 Apr 28;39(5):btad275. doi: 10.1093/bioinformatics/btad275 (PMC10185404; doi:10.1093/bioinformatics/btad275)
Supplement: btad275_Supplementary_Data [file btad275_supplementary_data.docx]

Supplementary Material for

**Predicting Allosteric Pockets in Protein Biological Assemblages**

Ambuj Kumar, Burak T. Kaynak, Karin S. Dorman, Pemra Doruker, Robert L. Jernigan

**Supplementary Table 1.** Ranks of known allosteric pockets obtained by APOP, Allopred, Passer and Fpocket for biological assembly structures. Active site pocket ranks by APOP are listed together with the structural details for each protein. It is important to note that ranking of active site pocket is obtained using APOP score of all pockets in the protein, whereas ranking of allosteric pocket pocket by APOP, Allopred, Passer, and Fpocket is conducted after removing active site pocket from the list in all the proteins listed in this table.

| **PDB ID** | **APOP active site pocket rank** | **Allosteric pocket rank** *^d^* | | | **Fpocket ranking of known allosteric pocket** *^e^* | **Total number of pockets** | **Total number of residues in protein** | **Number of chains** | **Ligand PDB ID** | **Name** |
| --- | --- | --- | --- | --- | --- | --- | --- | --- | --- | --- |
|  |  | **APOP allosteric pocket rank** | **Allopred allosteric pocket rank** | **Passer allosteric pocket rank** *^c^* |  |  |  |  |  |  |
| **2BU7** | 1 | 1 | 2 | 1 | 27 | 54 | 718 | 2 | TF3 | Pyruvate dehydrogenase kinase isozyme 2 |
| **4C7B** | 1 | 1 | 1 | 1 | 1 | 17 | 264 | 1 | BVB | NAD-dependent protein deacetylase sirtuin-3, mitochondrial |
| **3EPS** | 4 | 1 | 2 | 2 | 7 | 36 | 566 | 1 | AMP | Isocitrate dehydrogenase kinase/phosphatase |
| **3F6G** | 10 | 1 | 1 | 1 | 1 | 15 | 236 | 1 | ILE | Alpha-isopropyl malate synthase |
| **4EBW** | 7 | 1 | 1 | 1 | 1 | 16 | 259 | 1 | 0PF | Focal adhesion kinase 1 |
| **1QP0** | 2 | 1 | 1 | 1 | 1 | 35 | 676 | 2 | HPA | HTH-type transcriptional repressor PurR |
| **4MDK** | 23 | 1 | 1 | 1 | 1 | 26 | 244 | 1 | U94 | Ubiquitin-conjugating enzyme E2 R1 |
| **3O2M** | 1 | 1 | 17 | 2 | 11 | 25 | 368 | 1 | 46A | Mitogen-activated protein kinase 8 |
| **3H1V** | 3 | 1 | 1 | 1 | 21 | 21 | 446 | 1 | TK1 | Glucokinase |
| **3PXF** | 1 | 1 | 1 | 1 | 6 | 18 | 302 | 1 | 2AN | Cyclin-dependent kinase 2 |
| **3HO6** | 2 | 1 | 1 | 3 | 1 | 14 | 251 | 1 | IHP | Toxin A |
| **2JHR** | 20 | 1 | 1 | 1 | 4 | 48 | 776 | 1 | PBQ | Myosin-2 heavy chain |
| **2BXA** | 17 | 3 | 4 | NR | 2 | 39 | 569 | 1 | C1F | Serum albumin |
| **3R1R** | 3 | 3 | 20 | NR | 14 | 38 | 733 | 1 | ATP | Ribonucleoside-diphosphate reductase 1 |
| **3F9N** | 1 | 3 | 3 | 3 | 8 | 10 | 253 | 1 | 38M | Serine/threonine-protein kinase Chk1 |
| **3E5U** | 1 | 2 | 8 | 3 | 3 | 32 | 433 | 2 | 3C4 | Cyclic nucleotide-binding protein |
| **3MWB** | 17 | 5 | 3 | 1 | 2 | 64 | 1202 | 4 | PHE | Prephenate dehydratase |
| **3IFA** | 19 | 2 | 5 | 2 | 78 | 84 | 1294 | 4 | AMP | Fructose-1,6-bisphosphatase isozyme 2 |
| **1KZ8** | 12 | 6 | 2 | 2 | 2 | 69 | 1276 | 4 | PFE | Fructose-1,6-bisphosphatase isozyme 2 |
| **6ASC** | NA *^a^* | 1 | NA *^a^* | 1 | 1 | 39 | 652 | 2 | BU7 | Nuclease SbcCD subunit D |
| **1UXV** | 11 | 7 | 3 | 3 | 10 | 114 | 1996 | 4 | NAP | NAD(P)-dependent glyceraldehyde-3-phosphate dehydrogenase |
| **1FIY** | 161 | 1 | 1 | NR | 17 | 242 | 3492 | 4 | ASP | Phosphoenolpyruvate carboxylase |
| **1H9G** | 3 | 1 | 1 | 1 | 2 | 40 | 446 | 2 | MYR | Fatty acid metabolism regulator protein |
| **4JAF** | 1 | 16 | 28 | NR | 31 | 123 | 2556 | 6 | NAI | Citrate synthase |
| **1Z8D** | 42 | 1 | 3 | NR | 1 | 67 | 1638 | 2 | AMP | Glycogen phosphorylase, muscle form |
| **3N1V** | 22 | 3 | 8 | 3 | 12 | 53 | 686 | 2 | 3N1 | Farnesyl pyrophosphate synthase |
| **3H6O** | 6 | 1 | 6 | 1 | 117 | 121 | 1978 | 4 | D8G | Pyruvate kinase M2 |
| **5OJ0** | 3 | 1 | 2 | NR | 5 | 40 | 658 | 1 | 9WT | Penicillin-binding protein 2X |
| **1H5S** | 8 | 1 | 1 | NR | 2 | 64 | 1161 | 4 | TMP | Glucose-1-phosphate thymidylyl transferase 1 |
| **4BBG** | 4 | 1 | 2 | 1 | 3 | 63 | 1008 | 3 | V02 | Kinesin-like protein KIF11 |
| **1B86** | 1 | NP *^b^* | NP *^b^* | NR | 12 | 27 | 574 | 4 | DG2 | Hemoglobin |
| **1NH8** | 15 | NP *^b^* | NP *^b^* | NR | 8 | 36 | 552 | 2 | HIS | ATP phosphoribosyl transferase |
| **3PTZ** | 5 | 1 | 2 | 1 | 22 | 144 | 2744 | 6 | UDX | UDP-glucose 6-dehydrogenase |
| **2HVW** | 1 | 1 | 1 | NR | 2 | 37 | 878 | 3 | DCP | Putative deoxycytidylate deaminase |
| **1XTU** | 16 | 1 | 2 | 1 | 41 | 41 | 860 | 4 | CTP | Uracil phosphoribosyl transferase |
| **1FX2** | 6 | 5 | 4 | NR | 12 | 19 | 470 | 1 | DTT | Receptor-type adenylate cyclase GRESAG 4.1 |
| **4I1R** | 52 | 1 | 3 | 1 | 2 | 53 | 706 | 2 | LZU | Mucosa-associated lymphoid tissue lymphoma translocation protein 1 |
| **3PG9** | 61 | 4 | 3 | 1 | 2 | 76 | 1352 | 4 | TYR | Phospho-2-dehydro-3-deoxyheptonate aldolase |
| **3LAJ** | 16 | 1 | 1 | 1 | 4 | 33 | 911 | 6 | ARG | Arginine repressor |
| **4N9I** | 12 | 1 | 1 | 1 | 15 | 24 | 404 | 2 | PCG | Catabolite gene activator |
| **2YHD** | 2 | 2 | 3 | NR | 7 | 11 | 249 | 1 | AV6 | Androgen receptor |
| **3M3L** | 4 | 1 | 1 | 1 | 24 | 26 | 516 | 2 | P99 | Glutamate receptor 2 |
| **5LG3** | 14 | 1 | 1 | 1 | 89 | 92 | 1535 | 5 | Z80 | Gamma-aminobutyric-acid receptor subunit beta-1 |
| **5YVE** | 9 | 1 | 4 | 1 | 54 | 84 | 981 | 3 | AF9 | P2X purinoceptor 3 |
| **7F8H** | 7 | 1 | 1 | 1 | 1 | 14 | 259 | 1 | 1SI | Eyes absent homolog 2 |
| **6SAL** | 2 | 1 | 2 | 1 | 1 | 17 | 242 | 1 | L3E | Nuclear receptor ROR-gamma |
| **6T65** | 9 | 1 | 9 | 1 | 5 | 42 | 904 | 4 | MLH | 3-oxoacyl-(Acyl-carrier-protein) reductase |
| **5U2H** | 7 | 1 | 26 | 1 | 2 | 84 | 921 | 3 | 7S1 | P2X purinoceptor |
| **6VVQ** | NA *^a^* | 1 | NA *^a^* | 1 | 1 | 20 | 234 | 1 | MYR | Acyl-coenzyme A thioesterase 11 |
| **4MQT** | 6 | 1 | 1 | 1 | 22 | 25 | 396 | 1 | 2CU | Muscarinic acetylcholine receptor M2 |
| **Total** *^d^* | 9 (rank 1),  4 (rank 2),  4 (rank 3) | 35 (rank 1),  3 (rank 2),  4 (rank 3) | 19 (rank 1), 8 (rank 2),  7 (rank 3) | 29 (rank 1),  4 (rank 2),  5 (rank 3) | 11 (rank 1),  9 (rank 2),  2 (rank 3) |  |  |  |  |  |

*^a^* NA represents cases where active site residues are not resolved in the corresponding crystal structure. As Allopred requires this active site information for prediction, these two proteins cannot be assessed by Allopred.

*^b^* NP stands for no pocket. For Hemoglobin structure, Fpocket fails to determine allosteric ligand binding region as a pocket, and therefore APOP and Allopred fails to rank allosteric pocket.

*^c^* NR represents the allosteric pockets that are not ranked by Passer web interface. Passer web server only returns top three ranked pockets, and therefore NR is added when the allosteric pocket is not reported in the top three.

*^d^* We highlight known allosteric pockets predicted in the top three ranks by APOP, Allopred and Passer. The last row indicates the performance of each method based on predicting the top three ranking pockets. Here, active site pockets were removed from APOP, Allopred, and Passer ranking prior to ranking the known allosteric pocket.

*^e^* Original Fpocket ranking of known allosteric pockets are listed here. As Fpocket ranking is not aimed at allosteric pocket prediction, lower ranks are observed for many cases.

**Supplementary Table 2:** Ranking of known allosteric pocket in ligand bonded holo states and ligand free apo states for 12 proteins bfrom APOP.

| **Protein** | **Ligand bonded state** | | **Apo state** | | **Number of chains** | **Ligand** | **RMSD** |
| --- | --- | --- | --- | --- | --- | --- | --- |
|  | **PDB ID** | **rank** | **PDB ID** | **Rank** |  |  |  |
| TEM-1 Beta-Lactamase | 1PZO | 1 | 1ZG4 | Cryptic pocket | 1 | CBT |  |
| Protein Tyrosine Phosphatase 1B | 1T49 | 1 | 1OEM | 5 | 1 | 892 |  |
| Human glucokinase | 1V4S | 3 | 1V4T | 3 | 1 | MRK |  |
| Hepatits C NS5B RNA Polymerase | 3GNW | 2 | 4RY4 | 3 | 1 | XNC |  |
| Tryptophan synthase beta subunit | 2DH5 | 1 | 2DH6 | 1 | 1 | PLP |  |
| KSHV Protease | 4P3H | 1 | 1FL1 | 1 | 1 | 25G |  |
| Thymidylyltransferase | 1H5T | 1 | 1FZW | 1 | 4 | NVQ |  |
| Myosin-2 motor domain | 2JHR | 1 | 2Y0R | 1 | 1 | PBQ |  |
| Serine/threonine-protein kinase Chk1 | 3JVR | 3 | 1IA8 | 4 | 1 | AGX |  |
| Insulin-like growth factor 1 receptor | 3LW0 | 1 | 1P4O | 1 | 1 | CCX |  |
| Mitogen-activated protein kinase 8 | 3O2M | 1 | 3O17 | 1 | 1 | 46A |  |
| Cell division protein kinase 2 | 3PXZ | 1 | 1HCL | 7 | 1 | 2AN |  |
| Mitogen-activated protein kinase 14 | 4F9W | 1 | 5UOJ | 1 | 1 | LM4 |  |
| Uridylate kinase | 3EK5 | 1 | 3EK6 | 1 | 6 | GTP |  |
| HCV J4 RNA polymerase | 2NAD | 1 | 2NAC | 3 | 2 | NAD |  |
| **Total** |  | 12 (rank 1),  2 (rank 1),  1 (rank 3) |  | 9 (rank 1),  3 (rank 3) |  |  |  |

**Supplementary Table 3:** Ranking of known allosteric pockets in different structures of Pyruvate kinase assemblages

| **Protein** | **PDB ID** | **APOP rank of central allosteric activator pocket** |
| --- | --- | --- |
| Human pyruvate kinase M2 | 3H6O | 1 |
| Human pyruvate kinase M2 | 3BJF | 1 |
| Human pyruvate kinase M2 | 3ME3 | 1 |
| Human pyruvate kinase M2 | 3U2Z | 1 |
| Human pyruvate kinase M2 | 3GR4 | 1 |
| Human pyruvate kinase M2 | 4G1N | 1 |
| Trypanosoma cruzi pyruvate kinase | 4KRZ | 1 |
| Trypanosoma cruzi pyruvate kinase | 4KS0 | 1 |

**Supplementary Table 4:** Ranking of known allosteric ligand binding pockets in Tyrosine-protein phosphatase non-receptor type 1. RMSD values of each structure is measured against the structure with PDB ID 5KV9 to show the conformational differences between two structures.

| **Protein** | **Rank** | **Ligand** | **Conformational State** | **Mutation** | **RMSD** |
| --- | --- | --- | --- | --- | --- |
| 5K9W | 1 | TCS401 | Closed | None | 0.278 |
| 5K9V | 1 | None | Open | None | 0 |
| 5KA0 | 1 | None | Open | None | 0.092 |
| 5KA1 | 1 | TCS401 | Closed | None | 0.272 |
| 5KA2 | 1 | TCS401 | Open | Y152A, Y153A | 0.243 |
| 5KA3 | 1 | TCS401 | Closed | Y152A, Y153A | 0.268 |
| 5KA4 | 1 | None | Open | T178A | 0.272 |
| 5KA7 | 1 | TCS401 | Closed | T178A | 0.277 |
| 5KA8 | 1 | None | Open | L192A | 0.274 |
| 5KA9 | 1 | TCS401 | Open | L192A | 0.321 |
| 5KAA | 1 | None | Open | P185G | 0.138 |
| 5KAB | 5 | TCS401 | Open | P185G | 0.121 |
| 5KAC | 2 | None | Open | P185G | 0.116 |
| 5KAD | 1 | TCS401 | Closed | N193A | 0.376 |

**Supplementary Table 5:** Ranking of known allosteric ligand binding pockets in ABL kinase.

| **Protein** | **Rank** | **Ligand** |
| --- | --- | --- |
| 2V7A | 1 | PHA-739358 |
| 2HYY | 1 | Imatinib |
| 3K5V | 1 | Imatinib and GNF-2 |
